# Associations of Social Media Use With Physical Activity and Sleep Adequacy Among Adolescents: Cross-Sectional Survey

**DOI:** 10.2196/14290

**Published:** 2019-06-18

**Authors:** Sandhya V Shimoga, Erlyana Erlyana, Vida Rebello

**Affiliations:** 1 Department of Health Care Administration California State University Long Beach Long Beach, CA United States

**Keywords:** adolescent, social media, exercise, sleep

## Abstract

**Background:**

Adolescents’ use of social media, which has increased considerably in the past decade, has both positive and negative influences on adolescents’ health and health behaviors. As social media is the most prominent communication tool of choice for adolescents, it is important to understand the relationship between the frequency of social media use and health behaviors among this population.

**Objective:**

The objective of our study was to examine the associations between the frequency of social media use and physical activity and sleep adequacy among middle and high school students.

**Methods:**

We used data from the Monitoring the Future survey (2014 and 2015), a nationally representative, annual, cross-sectional survey of American 8th-, 10th-, and 12th-grade students (N=43,994). Health behaviors examined were frequency of vigorous physical activity and frequency of getting 7 hours of sleep (never/seldom, sometimes, and every day/nearly every day). We measured frequency of social media use using a Likert-like scale (never, a few times a year, 1-2 times a month, once a week, or every day). Multivariable generalized ordered logistic regressions examined the association of social media use with different levels of physical activity and sleep. We estimated marginal effects (MEs) for the main independent variable (social media use frequency) by holding all other variables at their observed values.

**Results:**

The study population comprised 51.13% (21,276/42,067) female students, 37.48% (17,160/43,994) from the South, and 80.07% (34,953/43,994) from a metropolitan area, with 76.90% (33,831/43,994) reporting using social media every day. Among physically active students, frequent social media use was associated with a higher likelihood of vigorous daily exercise (ME 50.1%, 95% CI 49.2%-51.0%). Among sedentary students, frequent social media use was associated with a lower likelihood of vigorous daily exercise (ME 15.8%, 95% CI 15.1%-16.4%). Moderately active students who used social media once or twice a month had the highest likelihood of reporting vigorous daily exercise (ME 42.0%, 95% CI 37.6%-46.3%). Among those who normally got adequate sleep, daily social media users were least likely to report adequate sleep (ME 41.3%, 95% CI 40.4%-42.1%). Among those who were usually sleep deprived, daily social media users were more likely to report adequate sleep (ME 18.3%, 95% CI 17.6%-19.0%).

**Conclusions:**

Regular social media use every day was associated with a reinforcement of health behaviors at both extremes of health behaviors, whereas a medium intensity of social media use was associated with the highest levels of physical activity and lowest sleep adequacy among those with moderate health behaviors. Hence, finding an optimal level of social media use that is beneficial to a variety of health behaviors would be most beneficial to adolescents who are in the middle of the health behavior spectrum.

## Introduction

### Background

Over the past decade, regular use of social media by young adults has increased considerably from 89% in 2014 to 97% in 2016 [1]. Villanti and colleagues also reported that young adults used an average of 7.6 social media sites regularly, with 85% of them using 6 or more sites regularly [1]. Adolescents’ time spent on social media has also more than doubled from 4.4 hours weekly in 2007 to 11.1 hours in 2011 [2].

Adolescents are more likely than any other age group to use social media. In 2013, approximately 45% of adolescents in the United States used social media sites daily and, among those, 73% used it to connect with their peers [3,4]. This extensive use of social media during the formative years calls into question the extent of its influence on all aspects of development, including physical and mental health. An emerging body of evidence shows both positive and negative influences of social media use on adolescents’ health and health behaviors [5-8]. The benefits of social media use include exposure to new ideas and information and raising awareness of current events and issues. The interactive nature of social media can provide opportunities to engage with peers on issues, access support networks, and improve social inclusion; it may also foster healthy eating habits [8]. However, there are notable negative health outcomes associated with social media use among adolescents. Higher levels of social media use among this population are associated with lower levels of participation in sports activity, less happiness, and more socioemotional difficulties [9]. Smartphone or mobile phone use at night is associated with reduced and disrupted sleep patterns among adolescents [6,10-12]. High-frequency social media use at night was significantly associated with perceived insufficient sleep [13]. While a high risk of poor-quality sleep or sleep disturbance is associated with frequent social media use among adolescents [11,12,14-16], there is some indication that sleeplessness precedes excessive media use [17,18]. Youths who connect with peers face-to-face have positive emotional outcomes compared with those who predominantly use social media to connect with peers [19,20]. Mental health conditions including anxiety and depression are exacerbated by excessive social media use among adolescents [15,21,22]. The predominant conclusion reached by studies on the relationship between social media use and health behaviors is that the time spent on social media use supplants time spent otherwise on physical activity or sleeping.

While many studies have focused on excessive use of social media or the internet, evidence is emerging that the relationship between intensity of internet use and health outcomes is not necessarily linear. Some recent studies suggested a U-shaped relationship between internet use frequency and depressive symptoms, where both low and high levels of internet use were associated with higher risks for depression and lower levels of mental well-being [21,23]. These studies examined cumulative use of all types of digital platforms. We did not find studies that specifically explored the relationship between frequency of social media use and health behaviors among adolescents.

### Objective

As social media is the most prominent communication tool of choice for adolescents, it is important to understand the relationship between the frequency of social media use and health behaviors among this population. Therefore, in this study, we examined the association between the frequency of social media use and different levels of physical activity and sleep adequacy among middle and high school students in the United States.

## Methods

### Data Source

Data for the study were from the Monitoring the Future (MTF) survey, which is a nationally representative, cross-sectional, annual survey of 8th-, 10th-, and 12th-grade students in the United States. MTF collects data through self-administered questionnaires in approximately 420 high schools and middle schools in the contiguous United States [24,25]. To obtain a nationally representative sample of schools and students, the survey employs a multistage random sampling design with (1) geographic areas or primary sampling units, (2) schools (or linked groups of schools) within primary sampling units, and (3) students within sampled schools. Schools are asked to participate for 2 years, and schools refusing participation are replaced with similar schools in terms of geographic location, size, and type of school. Student response rates for the survey were over 80% for all grade levels during all the years considered for our study and almost all nonresponse was due to absenteeism.

Data collection procedures were approved by the Institutional Review Board of the University of Michigan and the survey was funded by the National Institute on Drug Abuse. Public-use data files are available from Inter-university Consortium for Political and Social Research. Descriptions of the survey sampling frame, methodology, and response rates are detailed elsewhere [26].

We derived the study sample from MTF surveys conducted in 2014 and 2015. The data consisted of a pooled sample of 43,994 students in the 8th, 10th, and 12th grades who answered the questions on social media use and at least one of the questions on health behaviors. Eighth and 10th graders constituted 44.91% (20,011/43,994) and 45.99% (19,549/43,994) of the study sample, respectively. The questions pertaining to social media use and health behaviors were administered to approximately 20% of the 12th-grade students during 2014 and 2015 compared with 100% of the 8th- and 10th-grade students. Hence, 12th-grade students constituted a smaller portion of the study sample. We limited the selection of data to 2014 and 2015 due to major changes to the questions on social media use in 2014 [24,25].

### Dependent and Independent Variables

Our dependent variables were physical activity and sleep adequacy. The MTF survey measured responses to questions on physical activity (“How often do you exercise vigorously?”) and sleeping habits (“How often do you get at least seven hours of sleep?”) using a Likert-like scale with 5 possible responses: never, seldom, sometimes, most days, nearly every day, and every day. We created our dependent variables by combining adjacent categories of responses. We refer to the 3 levels of outcome variables as never/seldom, sometimes, and every day/nearly every day of physical activity or daily 7 hours of sleep.

We measured the main independent variable, frequency of social media use, by how often the student used social media (“How often do you visit social networking websites like Facebook, Twitter, Instagram, etc.?”) using a Likert-like scale with 5 possible responses: never, a few times a year, one to two times a month, once a week, or every day. We included the following individual level covariates in all our models: sex, average letter grade, number of hours of homework per week, grade level, and level of happiness on the day of the survey (very happy, pretty happy, or not too happy). We included school location (in a metropolitan statistical area as defined for the US Census or not) and the Census region in which the school was located to account for regional variations. We included survey year to account for differences between the survey years.

### Statistical Analysis

We used a repeated cross-sectional analysis by pooling survey data from the years 2014 and 2015 for 8th-, 10th-, and 12th-grade students. We employed multivariable generalized ordered logistic regressions to compare the association of social media use with different levels of physical activity and sleep adequacy. We used generalized ordered logistic models instead of ordered logistic regression models because the ordered logistic models did not meet the proportional odds assumption [27,28]. We tested for the joint significance of different levels of social media use using the adjusted Wald test. We estimated marginal effects (MEs) for the main independent variable, social media use frequency, by holding all other variables at their observed values. All analyses used pooled survey weights derived from the sampling frame sizes provided by Inter-university Consortium for Political and Social Research, along with the individual sample weights provided in the public-use data files. We conducted all analyses using Stata 14 (StataCorp LLC).

## Results

### Sample Characteristics

The study sample consisted of students who answered questions on social media use and questions on one or both of the health behaviors (N=43,994). Table 1 presents the characteristics of the study sample over different levels of social media use.

As Table 1 shows, the study population consisted of 51.13% (21,276/42,067) female students; 37.48% (17,160/43,994) were from the South and 80.07% (34,953/43,994) were from a metropolitan statistical area. Approximately half of the study population reported 1-4 hours of homework per week (21,029/42,332, 50.08%) and 75.25% (33,392/42,133) reported letter grades of B– or better. Most (28,381/43,460, 65.52%) of the students reported being pretty happy. Additionally 9.12% (4434/43,994) of the students were 12th graders, and 8th and 10th graders accounted equally for the rest of the study population.

Of the 20,879 students who answered both the questions on physical activity and on social media use, almost half (n=10,033) reported being physically active every day or nearly every day, while 17.06% (n=3563) reported lower levels of physical activity (Table 1). In all levels of social media use, a higher percentage of students reported being physically active regularly and a smaller percentage reported being sedentary.

Of the 20,950 students who answered both the questions on sleep and on social media use, about 41.82% (n=8689) reported getting 7 hours of sleep nearly every day, while 18.39% (n=3986) of the students reported never or seldom getting 7 hours of sleep (Table 1). In all levels of social media use except use every day, a higher percentage of students reported getting adequate sleep. Among those who used social media every day, an equal percentage of students reported getting moderately adequate (“sometimes”) or adequate levels (“every day/nearly every day”) of sleep.

**Table 1:** Characteristics of students by frequency of social media use.

Characteristics		Social media frequency observations, n (%)												*P* value			
		Never (n=3087)		A few times a year (n=990)		1-2 times a month (n=1607)		Once a week (n=4479)		Every day (n=33,831)		Total (N=43,994)					
**Frequency of physical activity**															<.001		
	Never/seldom		312 (22.3)		101 (21.0)		145 (19.0)		321 (16.7)		2684 (16.56)		3563 (17.14)				
	Sometimes		499 (36.8)		160 (37)		304 (40.5)		722 (36.1)		5598 (34.36)		7283 (34.98)				
	Every day/nearly every day		543 (40.9)		185 (42.0)		307 (40.5)		960 (47.2)		8038 (49.08)		10,033 (47.88)				
**Frequency of sleeping 7 hours/day**															<.001		
	Never/seldom		206 (13.8)		87 (20.0)		149 (19.2)		352 (16.3)		3192 (18.96)		3986 (18.39)				
	Sometimes		449 (33.1)		162 (36.0)		270 (34.5)		763 (38.7)		6631 (40.83)		8275 (39.79)				
	Every day/nearly every day		703 (53.1)		196 (44.0)		330 (46.3)		894 (45.0)		6566 (40.22)		8689 (41.82)				
**Survey year**															<.001		
	2014		1553 (50.00)		527 (53.0)		815 (49.7)		2256 (50.44)		15,935 (47.08)		21,086 (47.86)				
	2015		1534 (50.00)		463 (47.0)		792 (50.3)		2223 (49.56)		17,896 (52.92)		22,908 (52.14)				
**Grade**															<.001		
	8th		1811 (58.68)		511 (50.0)		754 (45.5)		2282 (50.79)		14,653 (42.68)		20,011 (44.91)				
	10th		1105 (36.56)		393 (42.4)		692 (45.3)		1780 (40.74)		15,579 (47.69)		19,549 (45.98)				
	12th		171 (4.7)		86 (8.0)		161 (9.0)		417 (8.5)		3599 (9.63)		4434 (9.11)				
**Sex**															<.001		
	Male		1989 (67.46)		670 (70.7)		1066 (70.13)		2786 (63.96)		14,280 (43.53)		20,791 (48.87)				
	Female		950 (32.5)		272 (29.3)		453 (29.9)		1454 (36.04)		18,147 (56.47)		21,276 (51.13)				
**Region**															<.001		
	North east		572 (16.2)		178 (17.1)		279 (15.0)		829 (17.0)		6631 (18.18)		8489 (17.78)				
	North central		604 (21.3)		204 (21.4)		311 (22.1)		912 (22.1)		6812 (22.00)		8843 (21.95)				
	South		1153 (35.8)		363 (35.1)		630 (36.9)		1657 (35.32)		13,357 (38.02)		17,160 (37.48)				
	West		758 (26.7)		245 (26.1)		387 (26.0)		1081 (25.61)		7031 (21.80)		9502 (22.79)				
**Metropolitan statistical area**															.01		
	No		597 (19.5)		222 (22.0)		364 (22.9)		995 (21.3)		6863 (19.59)		9041 (19.93)				
	Yes		2490 (80.5)		768 (78.0)		1243 (77.11)		3484 (78.74)		26,968 (80.41)		34,953 (80.07)				
**Level of happiness**															.03		
	Not too happy		502 (15.9)		159 (17)		285 (17.5)		650 (14.8)		5448 (16.34)		7044 (16.19)				
	Pretty happy		1918 (63.5)		634 (65.7)		1045 (66.14)		2945 (66.75)		21,839 (65.50)		28,381 (65.52)				
	Very happy		623 (20.6)		177 (17.8)		257 (16.4)		815 (18.5)		6163 (18.16)		8035 (18.29)				
**Homework (hours/week)**															.09		
	0		210 (7.3)		80 (9)		142 (8.7)		325 (8.09)		2693 (7.98)		3450 (7.99)				
	1-4		1397 (47.71)		462 (49.8)		773 (50.7)		2146 (51.08)		16,521 (50.14)		21,029 (50.08)				
	5-9		596 (20.1)		191 (20)		294 (19.3)		893 (20.6)		6362 (19.58)		8336 (19.72)				
	10-14		337 (11.2)		104 (10.9)		152 (10.1)		422 (9.5)		3327 (9.94)		4342 (9.99)				
	15-19		169 (5.6)		37 (4)		69 (5)		235 (5.1)		1781 (5.47)		2291 (5.39)				
	20-24		123 (4.7)		32 (4)		43 (3)		121 (2.8)		1174 (3.50)		1493 (3.49)				
	≥25		99 (3)		30 (3)		47 (3)		125 (2.8)		1090 (3.40)		1391 (3.34)				
**Letter grade**															<.001		
	D		82 (3)		33 (4)		58 (4)		102 (2.5)		856 (2.5)		1131 (2.63)				
	C–		89 (3)		32 (4)		68 (4)		147 (3.7)		1194 (3.69)		1530 (3.68)				
	C		117 (4.4)		48 (5)		92 (6)		278 (6.6)		1796 (5.55)		2331 (5.59)				
	C+		173 (5.6)		81 (9)		139 (9.3)		403 (9.9)		2953 (9.09)		3749 (8.93)				
	B–		203 (6.4)		102 (10.4)		160 (10.4)		455 (10.7)		3308 (10.33)		4228 (10.11)				
	B		374 (13.3)		148 (14.4)		236 (15.7)		698 (16.5)		4968 (15.16)		6424 (15.16)				
	B+		461 (15.4)		161 (17.3)		255 (18.5)		676 (15.6)		5725 (17.71)		7278 (17.35)				
	A–		592 (20.6)		156 (18.4)		231 (15.7)		724 (17.2)		5707 (17.49)		7410 (17.62)				
	A		835 (28.4)		177 (18.8)		256 (16.3)		771 (17.3)		6013 (18.42)		8052 (18.93)				

### Associations Between Dependent and Independent Variables

Results of multivariable regressions for physical activity indicated a statistically significant association between frequencies of social media use and physical activity (adjusted Wald test *F*_8,20871_=18.39; *P*<.001); and between frequencies of social media use and sleep adequacy (adjusted Wald test *F*_8,20942_=2.05; *P*=.04). Table 2 shows the MEs of different impacts of social media use on different levels of physical activity and sleep adequacy. We calculated the MEs holding all other covariates at their observed values.

Among students with a low level of physical activity, the likelihood of reporting physical activity decreased with increasing social media use (for social media nonusers, ME 26.4%, 95% CI 23.4%-29.4%, and for daily social media users, ME 15.8%, 95% CI 15.1%-16.4%). Among students with a higher level of physical activity, the likelihood of reporting physical activity increased with increasing frequency of social media use (for social media nonusers, ME 35.5%, 95% CI 32.6%-38.5%, and for daily social media users, ME 50.1%, 95% CI 49.2%-51.0%). In contrast to this linear relationship, the relationship among moderately active students peaked with students who used social media once or twice a month reporting the highest likelihood of vigorous exercise (ME 42.0%, 95% CI 37.6%-46.3%).

Among students who got inadequate sleep, social media nonusers were less likely to report getting 7 hours of sleep regularly (ME 16.6%, 95% CI 14.2%-18.9%) than were students who used social media every day (ME 18.3%, 95% CI 17.6%-19.0%). Among those who got adequate sleep, social media nonusers were more likely to report getting 7 hours of sleep (ME 46.6%, 95% CI 43.4%-49.7%) than were students who were daily social media users (ME 41.3%, 95% CI 40.4%-42.1%). Students who reported moderately adequate sleep and who used social media once or twice a month were least likely report adequate sleep (ME 36.4%, 95% CI 32.2%-40.6%).

Additional analysis including parental education and race/ethnicity as control variables did not significantly change the reported results. However, including these variables resulted in a smaller sample size, as one-third of the students did not provide parental education data and almost 29.92% (13,165/43,994) of the observations lacked data on race/ethnicity. Hence, we report the original results.

**Table 2:** Regression results showing the association between health behaviors and frequency of social media use by middle and high school students^a,b,c^.

Social media use frequency	% marginal effect (95% CI)					
	Vigorous physical activity (n=19,543)			Sleeping 7 hours/day (n=19,596)		
	Never/seldom	Sometimes	Every day	Never/seldom	Sometimes	Every day
Never	26.4 (23.4-29.4)	38.1 (34.8-41.3)	35.5 (32.6-38.5)	16.6 (14.2-18.9)	36.9 (33.7-40.0)	46.6 (43.4-49.7)
A few times a year	24.4 (19.8-28.9)	37.8 (32.4-43.2)	37.8 (32.5-43.1)	21.4 (16.9-25.9)	37.9 (32.6-43.3)	40.7 (35.4-46.0)
1-2 times a month	20.4 (16.9-24.0)	42.0 (37.6-46.3)	37.6 (33.6-41,6)	19.5 (16.2-22.9)	36.4 (32.2-40.6)	44.0 (39.9-48.2)
Once a week	18.6 (16.4-20.8)	36.5 (33.9-39.1)	44.9 (42.3-47.5)	17.4 (15.4-19.3)	40.1 (37.5-42.8)	42.5 (40.0-45.0)
Every day	15.8 (15.1-16.4)	34.1 (33.2-35.0)	50.1 (49.2-51.0)	18.3 (17.6-19.0)	40.4 (39.5-41.4)	41.3 (40.4-42.1)

^a^Generalized ordered logistic regression models are used for the estimation. Marginal effect indicates predicted percentage of students with different intensities of health behaviors and social media use, holding all the other covariates at their observed values.

^b^All results are statistically significant at *P*<.001.

^c^Data are from the Monitoring the Future survey (2014 and 2015) with students from 8th, 10th, and 12th grades.

## Discussion

### Principal Findings

Our study results indicate that the association between health behaviors and the frequency social media use is significant and the direction of the association differs by whether the health behaviors are at high, medium, or low levels. The health behaviors at both the high and the lower end of the spectrum are reinforced further with more frequent social media use, whereas moderate health behaviors show an interesting nonlinear relationship with the frequency of social media use. We adjusted for reported happiness to account for stress, which may adversely influence health behaviors. While having a higher homework load may take time away from physical activity, sleep, and social media use, we controlled for the homework hours in all models. We also adjusted for grade level, as students in higher grades may have less time for sleep and physical activities due to more academic demands on their time.

The results show that students who were moderately active (7283/20,879, 34.98% of the study sample) or who reported moderately adequate sleep (8275/20,950, 39.79% of the study sample) had a nonlinear relationship with the frequency of social media use. This relationship indicates that a moderate amount of social media use may have a beneficial impact on moderate physical activity and a detrimental influence on moderately adequate sleep among students. Most of the previous research did not delineate different levels of health behaviors, leading to the oversimplified conclusion that more social media use is associated with worse health behaviors. Our results are in line with the emerging literature on the relationship between social media use intensity and other health outcomes, including a U-shaped, nonlinear “Goldilocks effect” of the intensity of internet use on depression and overall mental well-being among adolescents [21,23]. The Goldilocks effect refers to the phenomenon where a moderate intensity of social media use is associated with the highest levels of mental well-being, compared with either too much or too little social media use.

These results are in line with the evidence that users extend their offline personality and behaviors into their online activities [29]. Our results indicate that, among physically active students, frequent social media use was associated with a higher likelihood of daily vigorous physical activity, which may be due to a positive feedback loop from sharing related news or pictures on social media. On the other hand, sedentary behaviors changed for the worse with increased social media use, which may further the supposition that time spent on social media is not available for other activities. Sleeping behaviors of both inadequate and adequate sleepers were reinforced with more frequent social media use. Inadequate sleepers may have had difficulty falling asleep or may have been light sleepers, and such issues were magnified with more social media use. On the other hand, adequate sleepers may have developed sleeping habits that were immune to social media use.

### Implications

These results have several implications for adolescents as their social media use has drastically increased over time. Adolescents’ social interactions have increasingly moved to social media platforms. To capitalize on this trend, there has been a marked shift in promoting health behaviors and providing interventions using social media platforms, which contribute to further increases in the frequency of social media use. While there is cause for concern, our study indicates some opportunities. There may be a “sweet spot” where a moderate level of social media use may provide maximum or minimum benefit for a particular health behavior for a specific set of students. The results also suggest that a one-type-fits-all approach may be detrimental to those who are already in the group that is at most risk of adverse behaviors.

While students may engage in vigorous physical activity due to participation in school teams and other organized activities, the levels of physical activity tend to decrease as they graduate from high school, go to college where activities are less structured, and eventually settle into adulthood, when they have to be self-motivated to remain physically active [30]. Unlike physical activity, sleep patterns tend to fluctuate more; however, most of the adult population falls into a moderately adequate sleep category with average reported sleep of less than 6 hours per day [31]. Further, average use of social media does not seem to decrease much as teenagers settle into early adulthood [4,32]. While the relationship between healthy behaviors and social media use in late adulthood may be influenced by additional factors, it is important to understand this nonlinear relationship during early adulthood, when many of the health behaviors are formed. Social media use is shaping up to be a large influencer of health behaviors, with social media-based programs being developed to improve fitness, reduce stress, share experiences on health behaviors, and form support groups to improve health and wellness for all age groups [7,33]. However, there is also growing evidence that social media may promote negative emotions due to excessive social comparison, including those on health behaviors [34-36]. Hence, it is imperative to understand whether there is an optimal level of social media use that has the best impact on health behaviors.

### Limitations

This was a cross-sectional study and, hence, we cannot claim to assess causality between health behaviors and social media use. The study population was a nationally representative sample of students. However, caution should be exercised when generalizing the results to other adolescents who are home schooled or who have dropped out of school.

While there was a survey question on the number of hours spent on social media, a large percentage of the answers were either missing or inconsistent and, hence, not used in this study. Since time used for social media was affecting health behaviors [6,11,13], future studies would benefit from including the actual time spent on social media activities rather than categories.

The data did not include information on specific social media sites visited or on specific activities conducted on social media (such as posting and responding to messages, or creating content). While results indicate reinforcement of both high and low levels of health behaviors, understanding the content and its specific methods of use by students would provide deeper insights into understanding the nonlinear relationship among those with moderate health behaviors. Inadequate sleep may be worsened further by social media content.

Students who exercise vigorously may be involved in organized sports activities such as school athletic programs or competitive sports outside the school and, hence, may be more disciplined in their commitment to physical activities. In that case, the effect of social media use on their physical activity levels may be overstated. We controlled for this to some extent by adjusting for academic achievement as a proxy for their intrinsic motivation. Students who sleep less may do so due to issues related to anxiety, depression, or other stressors, which may also influence use of social media. We accounted for this by including level of happiness as a proxy for mental well-being. The survey question on sleep asked about the regularity of getting 7 hours of sleep, whereas the US National Sleep Foundation and American Academy of Sleep Medicine recommend 8 to 10 hours of sleep for teenagers [37]. Hence, the survey possibly underrepresented the inadequacy of sleep. Future studies should include variables that capture sleep quality or problems in addition to questions on sleep duration.

### Conclusion

Our study indicates that regular social media use reinforces health behaviors of adolescents at the extreme ends of the health behavior spectrum. Among adolescents who follow health behaviors to a moderate extent, a moderate use of social media provides the most benefit. Future studies examining the content of social media sites visited and the interaction patterns, as well as the number of hours spent on social media sites, would inform our understanding of the impact of social media use on health behaviors and facilitate using optimal social media to promote health behaviors.

## Abbreviations

MTF: Monitoring the Future
ME: marginal effect
